# P16 The impact of socio-economic deprivation on vaccine hesitancy across pharmacies in Plymouth

**DOI:** 10.1093/jacamr/dlaf118.023

**Published:** 2025-07-14

**Authors:** Maha Murtaza, Shane McNerney, Finnegan Manley, Prasanth Neelam Naganathan, Yana Khatri, Nicholas Hobbs, Isabella Parker, Lotte Wilde

**Affiliations:** Faculty of Medicine and Dentistry, University of Plymouth, Plymouth, UK; Faculty of Medicine and Dentistry, University of Plymouth, Plymouth, UK; Faculty of Medicine and Dentistry, University of Plymouth, Plymouth, UK; Faculty of Medicine and Dentistry, University of Plymouth, Plymouth, UK; Faculty of Medicine and Dentistry, University of Plymouth, Plymouth, UK; Faculty of Medicine and Dentistry, University of Plymouth, Plymouth, UK; Faculty of Medicine and Dentistry, University of Plymouth, Plymouth, UK; Faculty of Medicine and Dentistry, University of Plymouth, Plymouth, UK

## Abstract

**Background:**

Plymouth has areas of varying levels of deprivation, as part of our study to understand the public knowledge of Anti-microbial resistance we sought after what influences affected vaccine hesitancy.

**Objectives:**

The study aims to explore the factors influencing vaccine hesitancy amongst Plymouth residents, to assess how differing socio-economic backgrounds and education status impact vaccine uptake.

**Methods:**

A total of 110 participants contributed to our survey based study across five pharmacies over two weeks (December 2024-January 2025) to assess the relationship between social deprivation and flu vaccine hesitancy. Survey data was compared with the 2021 national census deprivation map, which included factors like housing, health, education and unemployment.^1^

**Results:**

Out of 110 responses, 50 had already received the vaccine. Of the 60 remaining who hadn’t received their vaccine, 45 (75%) said they were not intending to get vaccinated. This meant that 40% of all participants were unwilling to receive their vaccines. Reasons for this hesitancy included lack of awareness about eligibility (11/45, 24.5%); lack of belief in the effectiveness (17/45, 37.8%); concerns about side effects (17/45, 37.8%); lack of necessity (2/45, 4.4%); time constraints (1/45, 2.2%); and no reasons stated (3/45, 6.6%). There were no significant variations in education levels across pharmacies, however deprivation rates varied across pharmacy locations: King Street(23%), St Levans(19.4%) and Devonport(21.7%) had similar deprivation rates. Mile house(15%) had a slightly lower rates and Hyde Park (10%) had the lowest deprivation rates. Our survey concluded that vaccine hesitancy rates correlated with these deprivation levels, with higher deprivation areas showing greater unwillingness to get vaccinated: Devonport (52.9%), King Street (48.7%), St. Levan (46.5%), Mile house (30%), and Hyde Park (11.1%).

**Conclusions:**

Despite our small sample, there is an association that was observed between higher deprivation rates and lower vaccine uptake, suggesting that socio-economic factors, rather than education levels alone, play a crucial role in vaccine hesitancy. This study shows a vital need to address public concerns and education about vaccine effectiveness and side effects to mitigate vaccine hesitancy in areas with high levels of deprivation.

## References

[1] Household Deprivation – Census Maps, ONS . Office for National Statistics. www.ons.gov.uk/census/maps/choropleth/population/household-deprivation/hh-deprivation/household-is-deprived-in-one-dimension

